# One Versus Two Screw Fixation in Minimally Invasive Hallux Valgus Surgery: A Systematic Review

**DOI:** 10.7759/cureus.97907

**Published:** 2025-11-27

**Authors:** Thomas L Lewis, Lily Fletcher, Ettore Vulcano, Tyler Gonzalez, Jonathan Kaplan, Choon Chiet Hong, Bradley P Abicht, Robbie Ray, Peter Lam

**Affiliations:** 1 Trauma and Orthopaedic Surgery, Royal National Orthopaedic Hospital NHS Trust, London, GBR; 2 Foot and Ankle Surgery, Orthopaedic and Arthritis Specialist Centre, Victoria, AUS; 3 Orthopaedic Surgery, Columbia University, Mount Sinai Medical Center, Miami, USA; 4 Orthopaedic Surgery, University of South Carolina, Columbia, USA; 5 Orthopaedics, Duke Orthopaedics, Duke University School of Medicine, Durham, USA; 6 Orthopaedic Surgery, National University Hospital of Singapore, Singapore, SGP; 7 Orthopaedics, Podiatry, and Sports Medicine, Emplify Health by Gundersen, La Crosse, USA; 8 Trauma and Orthopaedics, King's College Hospital NHS Foundation Trust, London, GBR

**Keywords:** biomechanical stability, bunion, dual screw, hallux valgus, hardware complications, metatarsal osteotomy, minimally invasive surgery, percutaneous fixation, screw fixation, single screw

## Abstract

Minimally invasive surgery (MIS) for hallux valgus has evolved significantly, with ongoing debate about optimal fixation methods. Fourth-generation techniques typically use two metatarsal screws; however, single screw fixation may reduce operative time, radiation exposure, and cost. This systematic review compares clinical outcomes, radiographic results, and complications between one- and two-screw metatarsal fixation in minimally invasive hallux valgus correction.

A systematic review following Preferred Reporting Items for Systematic Reviews and Meta-Analyses (PRISMA) guidelines was conducted across MEDLINE, EMBASE, PubMed, and Cochrane databases from inception to March 2025. Studies comparing single versus two screw fixation in minimally invasive hallux valgus surgery were included. Risk of bias was assessed using the Risk Of Bias in Non-randomized Studies of Interventions (ROBINS-I) tool. Primary outcomes included radiographic parameters (Hallux Valgus Angle (HVA), Intermetatarsal Angle (IMA), Distal Metatarsal Articular Angle (DMAA)) and clinical outcomes (American Orthopaedic Foot & Ankle Society Score (AOFAS), Visual Analog Scale (VAS), Manchester-Oxford Foot Questionnaire (MOXFQ)). Secondary outcomes included operative details, complications, and revision surgery rates.

Five studies met inclusion criteria: two clinical (n = 153 patients, 162 feet) and three biomechanical studies. Clinical studies showed comparable radiographic correction and patient-reported outcomes between fixation methods. Single screw fixation demonstrated significantly shorter operative time and reduced fluoroscopy exposure. Hardware-related complications requiring removal were higher in two-screw groups (32% vs. 3% in one study; 1.9% vs. 0% in another). Biomechanical studies revealed that single screw fixation may provide insufficient rotational stability, while two-screw configurations demonstrated improved construct stability.

Current evidence suggests that single screw fixation may reduce radiation exposure, surgical time, and hardware removal rates, but offers reduced biomechanical stability compared to a two-screw construct in patients undergoing minimally invasive hallux valgus surgery. However, the high risk of bias, limited comparative data, methodological heterogeneity, and relatively short follow-up periods in existing studies preclude definitive conclusions. Current evidence is insufficient to establish definitive recommendations, and fixation strategy should be individualised based on deformity characteristics and patient factors.

## Introduction and background

Hallux valgus is a common forefoot deformity causing pain, functional limitations, and decreased quality of life [1,2]. Surgical correction remains the definitive management for symptomatic cases, with over 100 different techniques described in the literature [3]. While traditional open procedures such as the Scarf and Akin osteotomy have been widely adopted with reliable outcomes, there has been growing interest in minimally invasive or percutaneous surgery (MIS) for hallux valgus correction over the past two decades [3-5].

Minimally invasive or percutaneous hallux valgus surgery has evolved considerably since its introduction in the early 1990s [6,7]. The technique has progressed through multiple generations, each addressing limitations of previous approaches by utilising different fixation methods (no fixation, K-wires, screws). The most recent fourth-generation approaches emphasise multiplanar correction of rotational deformity through stable fixation using two screws in a specific configuration [3,8-11]. These are commonly referred to as Minimally Invasive Chevron-Akin (MICA), Metaphyseal Extra-Articular Transverse and Akin Osteotomy (META), and Percutaneous Chevron-Akin (PECA).

Osteotomy stability is influenced by multiple factors, including osteotomy geometry (chevron versus transverse configuration), screw trajectory, configuration and positioning, bone quality, degree of metatarsal head displacement, and patient-specific factors such as activity level and compliance with weight-bearing restrictions. Osteotomy stability is important, as the three-dimensional reduction manoeuvre needed to correct the hallux valgus angle (HVA) and intermetatarsal angle (IMA) frequently requires large metatarsal head translation, necessitating stable fixation to maintain position whilst the bone consolidates and unites.

**Figure 1:**
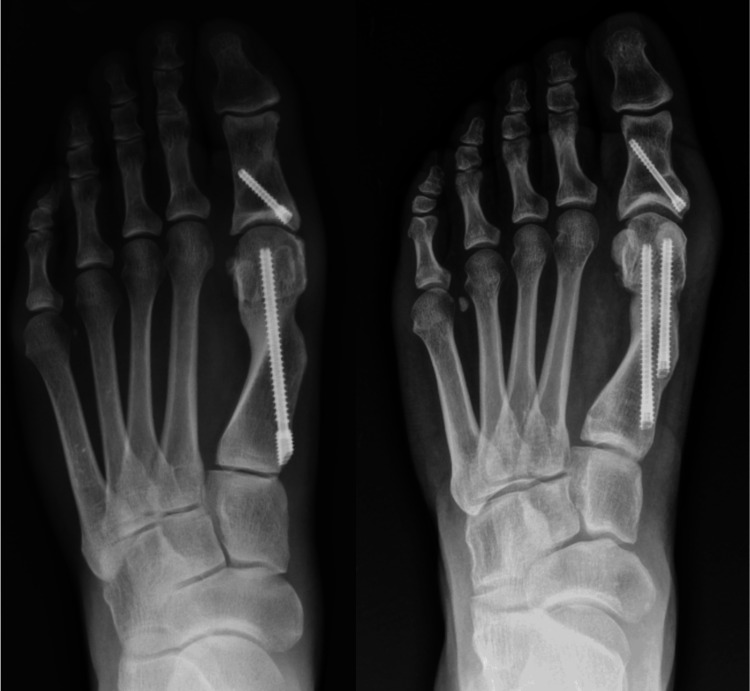
Radiographs demonstrating single-screw (left) and dual-screw (right) fixation following minimally invasive distal metatarsal osteotomy for hallux valgus correction. Radiographs courtesy of Dr. Ettore Vulcano and Dr. Peter Lam.

There remains significant debate regarding the optimal fixation method for minimally invasive hallux valgus surgery [12]. The original Redfern-Vernois technique for MICA utilised dual metatarsal screws to provide rotational stability and prevent displacement with early weightbearing, especially in cases with large metatarsal head translation [13,14]. However, studies have reported positive clinical and radiographic outcomes with single-screw fixation alone (Figure 1). Single-screw fixation may offer several advantages, such as reduced operative time, reduced fluoroscopy exposure, and lower cost, while potentially limiting complications such as hardware irritation, particularly from the distal screw used in dual-screw fixation cases [15]. Single-screw fixation may also reduce forefoot width by allowing a greater resection of the medial ledge. However, there may be disadvantages associated with single-screw fixation, including reduced rotational stability, an increased risk of lateral wall fracture, and osteotomy displacement [16,17]. It also remains to be seen whether there is a difference in bony healing and remodelling between single and dual screw constructs [18,19].

Objectives

This systematic review aims to evaluate and compare the clinical outcomes, radiographic deformity correction (HVA, IMA), and complication rates between single and dual metatarsal screw fixation techniques in minimally invasive hallux valgus surgery, supplemented by biomechanical evidence to clarify the mechanical principles underlying clinical observations.

## Review

Methods

Study Design

This study was performed according to the Preferred Reporting Items for Systematic Reviews (PRISMA) guidelines [20]. Details of the protocol for this systematic review were prospectively registered on PROSPERO [21].

Search Strategy

In August 2025, two independent reviewers (TL, LF) searched MEDLINE, EMBASE, PubMed, and Cochrane databases from inception to March 2025 using terms related to hallux valgus, MIS, and screw fixation. The full search string was: (hallux valgus OR bunion OR metatarsophalangeal deformity) AND (minimally invasive OR percutaneous OR MIS OR MICA OR Chevron OR Transverse) AND (screw OR fixation OR single screw OR dual screw OR double screw OR hardware OR osteotomy). Additional studies were identified through manual searching of reference lists of included articles and key American, British, and European orthopaedic and podiatric scientific literature. Any disagreements were resolved through discussion with a third reviewer (PL).

Participants

The inclusion criteria were patients who had undergone primary minimally invasive surgical intervention for hallux valgus with explicit description of either single or dual metatarsal screw fixation methods, including patients with all severity grades of hallux valgus deformity, without restrictions on age, gender, comorbidities, or bone quality. We included studies utilising minimally invasive distal metatarsal osteotomy hallux valgus surgery techniques (performed using a high-torque, low-speed burr) utilising screw fixation.

Study Criteria

The inclusion criteria comprised full-text studies in any language that reported clinical, radiological, or biomechanical outcomes following or investigating minimally invasive hallux valgus surgery comparing single versus dual metatarsal screw fixation. We included only comparative clinical or biomechanical studies with two arms that reported differences in outcomes between the two groups. Due to the limited number of clinical papers comparing single versus dual screws, we elected to include biomechanical studies to provide a more robust narrative synthesis of the best available current data for the benefit of readers. 

The following study designs were considered: randomized controlled trials and prospective or retrospective comparative observational cohort studies. Exclusion criteria included case reports and studies failing to specify postoperative clinical or radiographic outcomes. We excluded revision hallux valgus surgery, cases of degenerative disease of the first metatarsophalangeal joint, inflammatory arthropathy (e.g., rheumatoid arthritis), procedures incorporating fusion techniques (such as first metatarsophalangeal joint arthrodesis or the Lapidus procedure), and studies examining only open surgical techniques without a minimally invasive comparison. Adjunctive forefoot procedures (e.g., Akin osteotomy, lateral soft-tissue release, adductor hallucis release, sesamoid procedures) were not restricted when performed with either fixation method.

Data Extraction

Results from the database searches were collated and duplicates removed. Papers were screened using titles, abstracts, and inclusion/exclusion criteria to identify suitable studies. Full-text papers were reviewed for inclusion, and citations were screened to identify any additional studies. To minimise bias, data were blindly extracted by two authors (LF and TL) independently using a purpose-designed proforma. Disagreements were resolved by discussion with a senior author (PL).

Study characteristic data were extracted regarding: type of minimally invasive procedure, screw configuration details, patient demographics (age, sex, hallux valgus severity), clinical and patient-reported outcome measures, operative details (surgical time, fluoroscopy exposure), radiographic parameters, recurrence rates, complications, and cost analysis where available.

Variables

Study characteristics extracted included: study design, number of patients, age, gender distribution, hallux valgus severity classification, study location, and follow-up duration. Primary outcome measures included radiographic parameters (HVA, IMA, Distal Metatarsal Articular Angle (DMAA)) and clinical outcomes (American Orthopaedic Foot & Ankle Society Score (AOFAS), Visual Analog Scale (VAS), Manchester-Oxford Foot Questionnaire (MOXFQ) scores). Secondary outcomes included operative details (operative time, number of fluoroscopy images, radiation dose), complications (hardware-related issues, recurrence, nonunion, metatarsal fractures), revision surgery rates, and cost analysis.

Risk-of-Bias Assessment and Study Quality

The studies included in the analysis were assessed for bias within their methodology. Non-randomized trials were assessed using the Risk Of Bias in Non-randomized Studies of Interventions (ROBINS-I) tool [22]. The studies were independently assessed by two authors (LF and TL) and given an overall assessment rating, with any disagreements resolved through discussion with the senior author (PL). Formal quality assessment of biomechanical studies was not performed due to the marked heterogeneity of biomechanical study designs and the limited number of available studies; instead, a qualitative appraisal of methodological strengths and limitations is provided in the discussion.

Statistical Methods

All data analysis was conducted using Microsoft Excel. Clinical and methodological heterogeneity was assessed qualitatively by examining differences in patient populations (age, deformity severity, inclusion criteria), surgical techniques (osteotomy configuration, screw specifications, postoperative protocols), outcome measures (timing and type of assessments), and study methodologies (design, risk of bias, follow-up duration). Due to the heterogeneity of study designs, outcomes, and methodologies in the available literature, a systematic narrative synthesis was conducted rather than a formal meta-analysis. The synthesis followed a structured approach based on the Synthesis Without Meta-analysis (SWiM) guidelines [23]. Continuous outcomes were summarised using descriptive statistics, reporting ranges and central tendencies where available. For dichotomous outcomes, proportions and frequency data were extracted and compared qualitatively across studies. Methodological and clinical heterogeneity between studies was assessed and reported. Results were considered statistically significant at a p-value of <0.05.

Funding and Ethical Approval

There was no specific funding to support this study. Ethical approval was not required for this systematic review.

Results

Study Selection 

**Figure 2:**
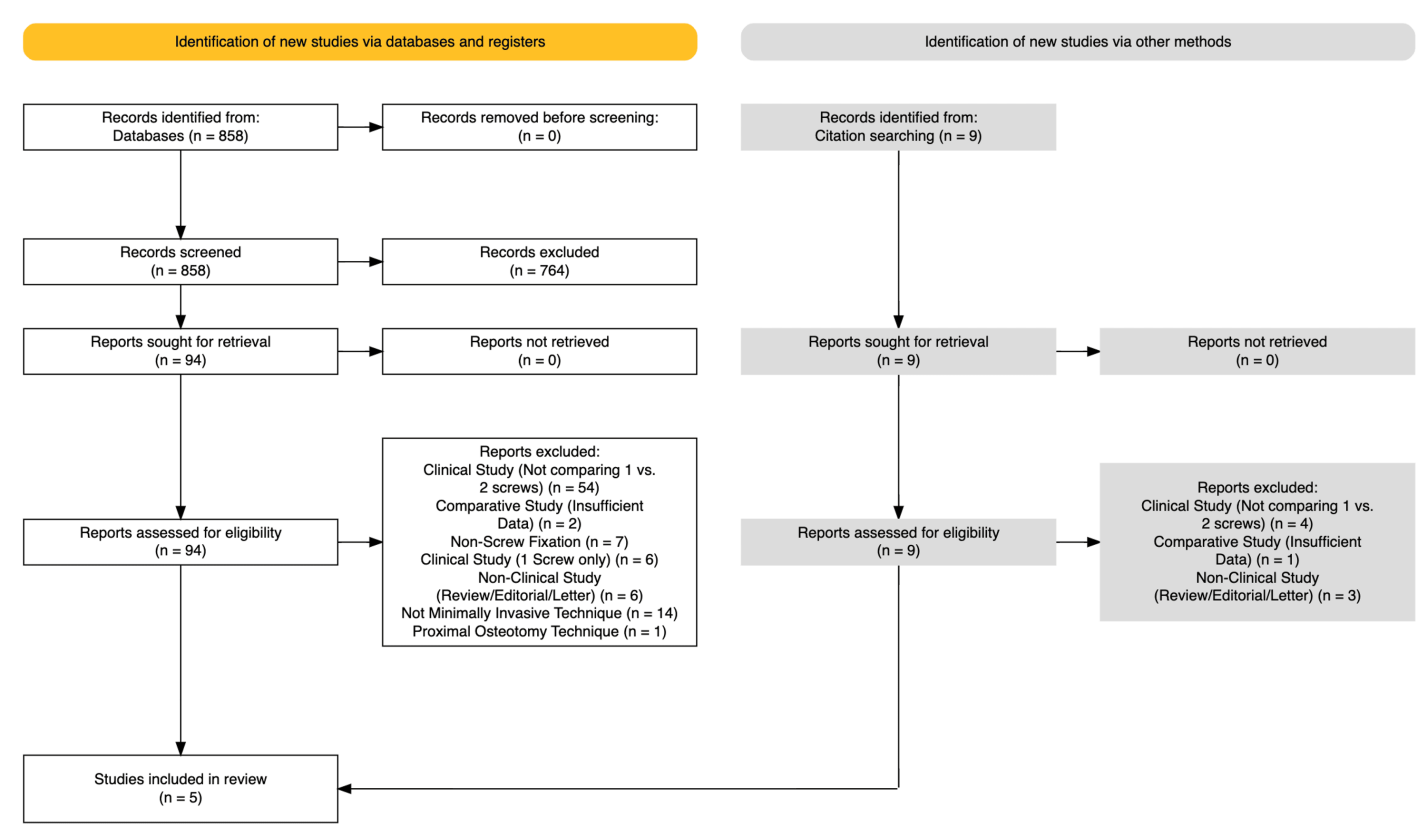
PRISMA flow diagram. PRISMA: Preferred Reporting Items for Systematic Reviews and Meta-Analyses.

A total of 858 potential studies were identified from the electronic database search, and 764 were excluded after reviewing their titles and abstracts. The remaining 94 full-text articles were reviewed, of which a further 90 studies were excluded. Finally, five studies were deemed suitable for inclusion following screening and assessment against all inclusion and exclusion criteria (Figure 2) [16,17,24-26].

Study and Patient Characteristics

The literature search identified two non-randomised observational studies and three biomechanical studies. The clinical studies included a total of 153 patients (162 feet) undergoing minimally invasive hallux valgus surgery with either single or dual screw fixation. Follow-up duration ranged from 12 to 21 months. Both clinical studies were retrospective in design, and all included studies were published between 2022 and 2024. The study characteristics, including postoperative protocols, are presented in Table 1. Two studies were excluded due to insufficient clinical data.

**Table 1:** Characteristics of included studies comparing different numbers of screw fixation for minimally invasive hallux valgus surgery. N.S.: Not specified.

Study	Type of Study	No. of Feet (patients; male:female)	Follow-up (months)	Age (yrs)	Country	Type of Osteotomy	Post-operative Protocol
Harrasser N et al. (2022) [24]	Retrospective comparative analysis of prospective data	55 (50; 7:43)	Min 12 m	1 Screw: 52 2 Screws: 45	Germany	Chevron	Bandaging for 2 weeks, then elastic taping to maintain hallux position. Mobilise full weight bearing as tolerated in a flat surgical shoe for 6 weeks.
Li X et al. (2022) [25]	Retrospective comparative	107 (103; 14:89)	21 ± 5	1 Screw: 43.9 ± 17.8 2 Screws: 44.6 ± 13.9	China	Chevron	Bandaging of hallux to maintain position for 6 weeks. Mobilise full weight bearing as tolerated in a flat surgical shoe for 4 weeks, then in a sports shoe.
Lewis TL et al. (2024) [17]	Biomechanical	1	N/A	46	Brazil	Chevron	-
Wagner P et al. (2024) [16]	Biomechanical	N/A	N/A	N/A	Chile	Transverse	-
Xu C et al. (2023) [26]	Biomechanical	2	N/A	N.S.	China	Transverse	-

Risk of Bias and Study Quality

**Figure 3:**
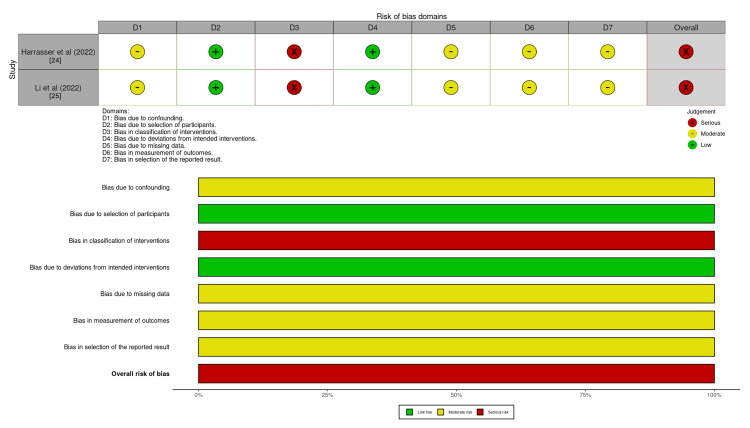
Risk of bias assessment of included clinical studies.

The studies included in the analysis were assessed for quality and risk of bias using the ROBINS-I tool (Figure 3) [22]. The main source of bias identified in the study by Harrasser N et al. was selection bias, as the MIS Chevron osteotomy was fixed with either one or two screws depending on the surgeon’s perception of intraoperative stability at the osteotomy site, potentially introducing a serious confounder [24]. Li X et al. excluded severe cases (HVA > 40° or IMA > 16°), introducing additional selection bias [25]. Neither study reported loss to follow-up or provided details of missing data or the number of excluded patients.

Radiographic Outcomes

Radiographic results demonstrated comparable correction of the HVA and IMA between single and dual screw fixation groups across both clinical studies (Table 2). The timing of radiographic assessment was 21 months in Li X et al.; however, the timing was not clearly stated in the study by Harrasser N et al., although it was likely at 12 months. No statistically significant differences were found between groups in final radiographic outcomes for either IMA or HVA in either clinical study (p > 0.05). Recurrence rates were low in both fixation groups. Harrasser N et al. reported recurrence in 3% (n = 1) of cases in the single-screw group and 5% (n = 1) in the dual-screw group [24]. Li X et al. observed recurrence rates of 5.7% and 3.7% in the single and dual screw groups, respectively, although the timing of recurrence assessment was not specifically reported [25]. Neither study identified statistically significant differences in recurrence rates between fixation methods. The nonunion rate was 0% across all patients in both clinical studies, regardless of fixation type.

**Table 2:** Radiographic deformity in clinical studies comparing different numbers of screw fixation for minimally invasive hallux valgus surgery. HVA: Hallux valgus angle; IMA: Intermetatarsal angle; DMAA: Distal metatarsal articular angle; N.S.: Not specified.

Study	Group (no. of pts)	Intermetatarsal Angle (IMA)		Hallux Valgus Angle (HVA)		Recurrence Rate	Non-union Rate	Other Radiographic Measures
		Pre-Operative	Post-Operative	Pre-Operative	Post-Operative	(HVA > 20° and/or IMA > 10°)		
Harrasser N et al. (2022) [24]	1 Screw (30)	15	5.5	30.9	10.2	N=1, 3%	0.00%	N.S.
	2 Screws (20)	14.6	5.8	30.8	11	N=1, 5%	0.00%	N.S.
Li X et al. (2022) [25]	1 Screw (51)	10.5±3.0	4.7±2.6	34.0±6.9	10.3±5.1	5.70%	0.00%	DMAA 14.0±4.1 to 4.9±3.5
	2 Screws (52)	10.5±3.0	3.9±2.0	34.2±8.8	10.1±5.2	3.70%	0.00%	DMAA 16.2±6.1 to 5.7±4.2

Clinical Outcomes

Patient-reported outcome measures were reported only in one study, by Li X et al., which demonstrated significant improvement from preoperative to postoperative assessments in both fixation groups (Table 3) for MOXFQ, AOFAS, and VAS pain scores [25]. No statistically significant differences were found between groups for any clinical outcome measure (p > 0.05 for all comparisons).

**Table 3:** Patient-reported outcome measures in clinical studies comparing different numbers of screw fixation for minimally invasive hallux valgus surgery. MOXFQ: Manchester-Oxford Foot Questionnaire; AOFAS: American Orthopaedic Foot and Ankle Society; VAS: Visual Analogue Scale; MTPJ: Metatarsophalangeal joint; N.S.: Not specified.

Study	Group (no of pts)	MOXFQ		AOFAS		VAS Pain		Complications
		Pre-Operative	Post-Operative	Pre-Operative	Post-Operative	Pre-Operative	Post-Operative	
Harrasser N et al (2022) [24]	1 Screw (30)	N.S.		N.S.		N.S.		Delayed wound healing: n = 3 (9%) Asymptomatic loss of correction: n = 1 (3%) Prominent screw: n = 1 (3%)
	2 Screws (20)							Delayed wound healing: n = 3 (14%) Deep infection: n = 1 (5%) Asymptomatic loss of correction: n = 1 (5%) Prominent screw: n = 7 (32%)
Li X et al (2022) [25]	1 Screw (51)	50.1±13.3	12.9±8.3	56.5±10.3	54.9±11.7	5.8±1.6	0.6±0.6	First MTPJ stiffness: n = 2 (3.8%) Numbness post-procedure: n = 2 (3.8%)
	2 Screws (52)	51.9±13.1	13.2±11.0	90.3±6.2	89.2±8.6	5.2±1.4	0.7±0.6	First MTPJ stiffness: n = 3 (5.6%) Numbness post-procedure: n = 3 (5.6%) Persistent dysaesthesia: n = 1 (1.9%) Prominent screw: n = 1 (1.9%)

Operative parameters favored single screw fixation, with Li X et al. reporting shorter mean operative time (46.9 ± 5.2 minutes vs. 56.6 ± 5.2 minutes, p < 0.001) and fewer intraoperative fluoroscopic images (15.4 ± 3.0 vs. 18.7 ± 2.1, p < 0.001) compared to dual screw fixation [25].

Complications

Complication rates were generally similar between the two groups. First metatarsophalangeal joint stiffness was observed in 3.8% (n = 2) of single-screw cases and 5.6% (n = 3) of dual-screw cases in the study by Li X et al., and at similar rates in the study by Harrasser N et al. [24,25]. Postoperative numbness was reported in 3.8% (n = 2) of single-screw cases and 5.6% (n = 3) of dual-screw cases by Li X et al. [25].

The largest difference between fixation methods was in hardware-related complications. Harrasser N et al. reported that 32% (n = 7) of patients in the dual-screw group had prominent screws requiring removal, compared to only 3% (n = 1) in the single-screw group [24]. Similarly, Li X et al. found that 1.9% (n = 1) of patients in the dual-screw group required hardware removal due to prominence, while no such complications were observed in the single-screw group [25]. No fractures were reported in either study.

Delayed wound healing occurred at similar rates in both fixation groups, with Harrasser N et al. reporting 9% (n = 3) in the single-screw group and 14% (n = 3) in the dual-screw group. One case of deep infection (5%) was observed in the dual-screw group by Harrasser N et al., with no infections reported in the single-screw group [24].

Biomechanical Outcomes

Three biomechanical studies were identified, including two finite element analysis studies and one sawbone model specifically examining single versus dual screw fixation configurations.

Lewis TL et al. conducted a finite element analysis using a model developed from CT images of a 46-year-old female with moderate hallux valgus deformity [17]. Five screw configurations were tested: a single bicortical screw, a single intramedullary screw, dual bicortical screws, dual intramedullary screws, and one bicortical plus one intramedullary screw (standard MICA configuration). This study found that the MICA configuration demonstrated the lowest osteotomy displacement compared to single intramedullary or bicortical screws. Stress analysis showed that the MICA configuration had the lowest maximum principal stress values, while the single bicortical screw configuration showed the highest tensile stress values.

Xu C et al. evaluated the mechanical properties of absorbable screws for fixation following distal transverse metatarsal osteotomy in hallux valgus using finite element analysis [26]. Two HV models with HVA of 30° and 40° were fixed with either one 4.5-mm absorbable screw or two 2.7-mm absorbable screws following osteotomy. They found that both fixation methods provided satisfactory mechanical performance (calculated stress limits were within the safe range to avoid implant failure under all conditions). There was no significant difference between the two fixation methods, although this study did not report key postoperative deformity data.

Wagner P et al. conducted a biomechanical evaluation comparing six different fixation configurations for percutaneous extracapsular transverse cervical metatarsal (PTCM) osteotomy in a hallux valgus sawbone model [16]. The authors utilised 30 solid foam sawbone foot models to assess the mechanical performance of six screw configurations under both cyclic and load-to-failure testing in a cantilever setup.

In their experimental design, specimens were divided into six groups: (1) A single 4.0-mm screw; (2) Two parallel screws (4.0 mm and 3.0 mm); (3) Two divergent screws (4.0 mm and 3.0 mm); (4) Two divergent screws with lateral metatarsal head cortex purchase; (5) Same as Group 4 but with two 4.0-mm screws; and (6) Same as Group 5 but with two 3.5-mm screws. 

They found that single-screw fixation (Group 1) was insufficient, failing under cyclic testing due to rotational instability. All two-screw configurations completed cyclic testing without failure, though stiffness differed significantly between groups. Using two 4.0-mm screws (Group 5) did not significantly improve stability compared with the 4.0-mm/3.0-mm combination in Group 4 (p = 0.1).

The mode of failure also differed among groups: Groups 2-3 failed primarily through implant loosening, whereas Groups 4-6 failed through proximal metatarsal fracture, indicating that the construct had become stronger than the bone itself.

Discussion

The available evidence, although limited and subject to a high risk of bias, provides initial guidance for clinical decision-making regarding screw fixation strategies, demonstrating that both fixation techniques can provide satisfactory clinical and radiographic outcomes in appropriately selected patients.

Clinical Findings

Whilst acknowledging the high risk of selection and reporting bias, both comparative studies demonstrated equivalent union rates and radiographic deformity correction, with no significant differences noted between the single and dual screw fixation groups. The equivalent union rates across fixation methods suggest that osteotomy healing is not primarily dependent on the number of fixation points but rather on adequate bony apposition and appropriate postoperative protocols, although concerns remain regarding displacement of the osteotomy with single-screw fixation, as this was not adequately reported. Hardware-related complications appeared more prevalent in the dual-screw groups. Harrasser N et al. reported that 32% of patients required screw removal in the dual-screw group, compared to only 3% in the single-screw group; however, this rate is much higher than that reported in other studies on percutaneous hallux valgus surgery [3,8,15,24]. Similarly, Li X et al. found that prominent hardware requiring removal occurred in 1.9% of dual-screw fixations but was not observed in the single-screw group [25]. Reduced operative time, cost, and fluoroscopy exposure were also reported with single-screw fixation.

Biomechanical Evidence

The biomechanical evidence comparing single and dual screw fixation includes three studies using different methodologies. Lewis T et al. found that constructs using one bicortical and one intramedullary screw demonstrated optimal performance, with the lowest osteotomy displacement and most favourable stress distribution [17]. Wagner P et al., using sawbone testing of six different screw configurations under cyclic and load-to-failure conditions, found that single-screw fixation consistently failed due to rotational instability [16]. Xu C et al. found no significant difference between single and dual screw fixation methods, although this study had several limitations, including the lack of specific reporting on postoperative correction [26].

Several studies outside the scope of this review have reported on single-screw fixation for minimally invasive hallux valgus surgery without comparative groups [27-34]. These studies generally show positive clinical and radiographic outcomes with low complication rates. However, these findings must be considered alongside the much larger body of evidence supporting dual-screw fixation as described in the original third-generation MIS technique by Redfern and Vernois [13,35-40].

The literature on dual-screw fixation spans multiple studies with longer follow-up periods and larger patient cohorts, providing a more robust foundation for its use [5,15,41]. This imbalance in the evidence base makes it difficult to draw definitive conclusions about the superiority of either approach, given the lack of randomised or methodologically robust comparative data.

As summarised in Table 4, the trade-offs between fixation methods involve multiple considerations beyond biomechanical stability. The practical advantages of single-screw fixation, reduced radiation exposure, shorter operative time, potentially lower cost, and fewer hardware-related complications, must be balanced against concerns regarding rotational stability, osteotomy displacement (particularly in severe deformity or poor bone quality), and the limited long-term evidence base.

**Table 4:** Advantages and disadvantages of single versus dual screw fixation in minimally invasive hallux valgus surgery. Original table prepared by the authors. IMA: Intermetatarsal Angle.

Parameter	Single Screw Fixation	Dual Screw Fixation
Operative Efficiency	Reduced operative time; shorter duration	Increased operative time compared with single-screw technique
Radiation Exposure	Decreased fluoroscopy exposure; fewer intraoperative images required	Greater radiation exposure; more intraoperative fluoroscopy required
Cost	Lower cost; elimination of second screw reduces implant cost	Higher cost; additional screw increases procedural cost and operative time
Hardware Complications	Potentially reduced hardware-related complications; lower rates of symptomatic hardware (3% vs. 32%) requiring removal	Higher rate of hardware-related complications from distal screw
Foot Width	Potentially reduced foot width as a larger medial prominence can be resected compared with dual-screw fixation	Medial ledge resection limited by location of the distal screw
Coronal Stability	Reduced stability; may provide less rotational control, particularly for severe deformities with >100% translation	Enhanced biomechanical stability; two points of fixation providing rotational control and coronal stability
Rotational Stability	Concerns about osteotomy displacement; theoretical risk of rotational instability	Second screw prevents rotation of the capital fragment
Clinical Evidence	Limited evidence on long-term follow-up; comparative studies report only short- to medium-term outcomes (12-24 months)	More extensive clinical evidence; greater number of published studies with medium- to long-term outcomes
Technical Complexity	Potentially less technical complexity; may be beneficial during learning curve	Potentially more technically demanding to place the second screw appropriately
Bone Healing	Limited number of studies; however, no clear concerns regarding bone healing	More extensive clinical evidence; very low rate of non-union
Applicability to Severe Deformity	Limited evidence supporting use in severe hallux valgus (>40° HVA, >16° IMA)	Potentially better suited for severe deformities; may provide more reliable fixation for large (>100%) lateral translations
Fracture Risk	Increased risk of lateral cortex fracture due to higher stress at the lateral cortex	Risk of lateral cortex fracture; placing two screws close together with a small cortical bridge can weaken the cortex
Early Weightbearing	May require delayed weightbearing in some cases, particularly with poor bone quality or large head shift	Enhanced stability may facilitate earlier weightbearing protocols

Limitations

This systematic review has several limitations that should be acknowledged. First, the small number of included clinical studies (n = 2) limits the strength of our conclusions; however, this review summarises the comparative literature available. Second, the included clinical studies were retrospective in nature and subject to selection bias. It is possible that patients assigned to single-screw fixation had less severe deformities, improved stability, or better bone quality, potentially confounding the results. Li X et al. specifically noted that their study was limited to mild and moderate hallux valgus (IMA < 16°), making it unclear how these findings may apply to more severe deformities [25]. The osteotomy configuration may also have an impact on overall construct stability, and these findings may not be generalisable to other osteotomies such as the transverse osteotomy, which is currently widely utilised [8,11].

Third, follow-up periods in the clinical studies were relatively short (12-21 months), which may be insufficient to detect long-term differences in outcomes, particularly regarding recurrence rates [42].

Fourth, the biomechanical studies used different methodologies (finite element analysis versus sawbone testing) with varying loading conditions that may not accurately represent physiological forces experienced during activities of daily living. The study by Wagner P et al. utilised synthetic sawbone models, which may not fully replicate the mechanical properties of human bone compared with cadaveric studies, potentially limiting the clinical applicability of these biomechanical findings [16]. The translation of these biomechanical findings to clinical outcomes remains uncertain.

The limitations of the current evidence base highlight the need for high-quality, randomised controlled trials comparing single and dual screw fixation techniques. Future studies should include larger sample sizes, confidence intervals, effect sizes, longer follow-up periods, and stratification by deformity severity and patient characteristics. Additionally, standardised reporting of surgical techniques and complications [43,11], including osteotomy configuration and screw parameters, would facilitate comparisons across studies and provide robust methodological evidence.

Economic analyses are also needed to evaluate the cost-effectiveness of different fixation strategies, accounting for both direct costs (implants, operating time) and indirect costs (complications, reoperations). The potential reduction in hardware-related complications with single-screw fixation may translate into significant cost savings and improved patient satisfaction.

## Conclusions

Current evidence suggests that single-screw fixation may reduce radiation exposure, surgical time, and hardware removal rates but may offer reduced biomechanical stability compared with a two-screw construct in patients undergoing minimally invasive hallux valgus surgery. However, the high risk of bias, limited comparative data, methodological heterogeneity, and relatively short follow-up periods in existing studies preclude definitive recommendations. Surgeons should consider individual patient factors, deformity characteristics, and their own technical expertise when selecting between fixation strategies. Further high-quality comparative research is needed to better define the optimal approach for different clinical scenarios.
